# Effect of Probucol and Atorvastatin Treatment on the Self‐Care Ability of Acute Cerebral Infarction Patients: A Randomized Clinical Trial Study

**DOI:** 10.1002/brb3.70552

**Published:** 2025-05-13

**Authors:** Ning Yu, Yajing Li, Xue Han, Yuan Sun, Yun Li, Yanjun Gao, Xiaoxuan Zhang

**Affiliations:** ^1^ Department of Neurology Affiliated Hospital of Chengde Medical University Chengde Hebei Province China

**Keywords:** acute cerebral infarction, atorvastatin, probucol, self‐care ability

## Abstract

**Background:**

To investigate the effect of probucol and atorvastatin on self‐care ability in patients with acute cerebral infarction (ACI).

**Methods:**

Eighty‐one patients with ACI admitted from November 2020 to May 2021 were divided into a combination treatment group (n = 40) and an atorvastatin group (n = 41). The atorvastatin group was given atorvastatin on the basis of conventional treatment, and the combination treatment group was treated with probucol and atorvastatin on the control basis. Self‐care ability and blood lipid levels were assessed at baseline and 6 months after treatment using the Barthel Index (BI) and biochemical tests. The study also included MRI scans to evaluate infarct volume and monitored relevant clinical indicators.

**Results:**

The baseline data of both groups were consistent and comparable. After six months, both groups showed improved self‐care ability, with the combined treatment group exhibiting a more significant improvement (P < 0.05). The combination treatment group also demonstrated a significant reduction in total cholesterol (TCH) and low‐density lipoprotein (LDL) levels compared to the atorvastatin group (P < 0.05). The factors influencing self‐care ability included history of cerebrovascular disease, treatment modality, and LDL levels.

**Conclusion:**

Probucol combined with atorvastatin significantly enhances self‐care ability and improves blood lipid levels in patients with ACI. This combination therapy is safe, feasible, and recommended for clinical application to reduce cognitive decline and improve quality of life in these patients.

## Graphical abstract

This study assessed the effects of combining probucol with atorvastatin on self‐care ability and blood lipids in acute cerebral infarction (ACI) patients. Eighty‐one patients within 72 h of onset were assigned to receive either atorvastatin alone or atorvastatin plus probucol. Key measures included blood lipid levels, Barthel Index (BI) for self‐care, and MRI for infarct volume. The combination group showed greater reductions in total cholesterol and LDL, with improved BI scores after 6 months. These results suggest that probucol combined with atorvastatin enhances lipid control and self‐care in ACI patients.

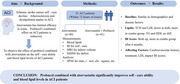

## 1 Introduction

Stroke includes three subtypes: ischemic stroke (IS), non‐traumatic intracranial hemorrhage, and spontaneous subarachnoid hemorrhage. Among them, ischemic stroke is the most common, which is the third cause of global disease burden (GBDS Collaborators 2021). Stroke not only leads to a significant loss of life but also imposes a considerable societal burden on survivors as their ability to take care of themselves declines (Palmer et al. 2019; Rost et al. 2022). Therefore, how to improve the self‐care ability of stroke survivors is a topic worthy of discussion.

Atherosclerosis is a well‐known cause of cardiovascular disease and stroke; research has indicated that atherosclerosis (AS) and dyslipidemia play significant roles in the pathophysiology of acute cerebral infarction (ACI) (Ni et al. 2020). The management of ACI plays a crucial role in alleviating the economic burden associated with stroke, particularly in economically disadvantaged regions (Herrington et al. 2016). Simultaneously, AS can elevate the likelihood of developing hyperlipidemia. AS and hyperlipidaemia may trigger and affect each other, respectively. Currently, ACI is predominantly managed with medications, including atorvastatin, a common ACI treatment drug and widely utilized lipid‐regulating medication. Atorvastatin can effectively lower lipids, exert anti‐inflammatory effects, and promote the stability of AS plaque, thereby improving the ACI condition and reducing nerve damage (Chen et al. 2020). While rhabdomyolysis and liver function damage are uncommon, the risk of experiencing negative effects from statins is worrisome. However, researchers had declared that these adverse effects does not overshadow their effectiveness in preventing such diseases (Cai et al. 2021). Nevertheless, there are certain individuals suffering from ACI who do not experience effective relief with atorvastatin treatment. They may benefit from non‐statin lipid‐lowering therapy or combination therapy (Beshir et al. 2021). Consequently, they continue to be at risk of AS formation or rupture, which heightens the likelihood of recurrence. An alternative solution is the utilization of probucol, a novel lipid‐lowering medication that possesses antioxidant properties. This drug can impede cholesterol formation, decrease low‐density lipoprotein synthesis within the body, and effectively lower plasma oxygen free radical levels. Additionally, probucol exhibits a positive impact on AS, safeguarding nerve function against oxidative harm (Santos et al. 2020). Research has demonstrated that when probucol is combined with atorvastatin in the treatment of ACI, it enhances the positive impact of drug therapy on AS and effectively stabilizes vulnerable AS plaque (Guo et al. 2015). Despite these promising findings, the specific effects of this combination on self‐care ability and overall quality of life in ACI patients remain unclear. Therefore, this study aimed to observe the effects of probucol and atorvastatin in treating patients with acute ischemic stroke, monitor changes in relevant indicators and prognosis, and provide a scientific foundation for clinical treatment of this condition.

## 2 Material and Methods

### 2.1 Population

From November 2020 to May 2021, the Affiliated Hospital of Chengde Medical University conducted a study involving 81 patients with ACI who were admitted within 72 h of disease onset. The patients underwent either magnetic resonance imaging (MRI) or computed tomography.

To be included in the study, patients had to meet the following criteria: (1) have a new cerebral infarction (either the first episode or a previous history with a modified Rankin Scale score of less than 2 points); (2) meet the diagnostic criteria for AIS and be confirmed as having atherosclerotic cerebral infarction; and (3) voluntarily sign the informed consent form and be willing to cooperate. Patients who met any of the following criteria were excluded from the study: (1) a previous history of intracranial space‐occupying diseases, intracranial arteritis, or hypertensive encephalopathy; (2) presence of malignant tumors or other end‐stage diseases with an expected survival time of less than 1 year; (3) abnormal electrocardiogram, serious infection or inflammation, severe liver and kidney dysfunction, severe anemia, or severe coagulation dysfunction; (4) pregnant or lactating women; (5) patients who had been treated before admission and had taken lipid‐adjusted drugs within one month; (6) patients with severe dementia or severe mental disorders who were unable to undergo cognitive assessment; (7) drug allergy; and (8) contraindications to MRI.

The patients who met the enrollment criteria and signed an informed consent form were randomly distributed into combination treatment groups using the random shuffle module from Python 3.9.7 software. The study was approved by the Ethics Committee of the Affiliated Hospital of Chengde Medical College (approval number: LL047) and registered with the Chinese Clinical Trial Registry (ChiCTR2000040461).

### 2.2 Study Design and Treatment Plan

Upon admission, the patients received routine treatment, which included medications for antiplatelet aggregation, brain protection, free radical clearance, and blood pressure control. The atorvastatin group (n = 41) was administered atorvastatin in addition to the conventional treatment. Atorvastatin tablets (trade name: Ara; specification: 20 mg, 7 tablets; Chinese medicine approved H20093819; Beijing Jialin Pharmaceutical Co., Ltd.) were given at a daily dose of 20 mg without interruption for six months. The combination treatment group (n = 40) received both atorvastatin and probucol in addition to the conventional treatment. Probucol (trade name: Changtai; specification: 0.125 g, 8 tablets; Chinese medicine quasi‐number H10960161; Chengde Jingfukang Pharmaceutical Group Co., Ltd.) was administered at a dose of 500 mg twice a day (morning and evening) without interruption for six months.

**FIGURE 1:**
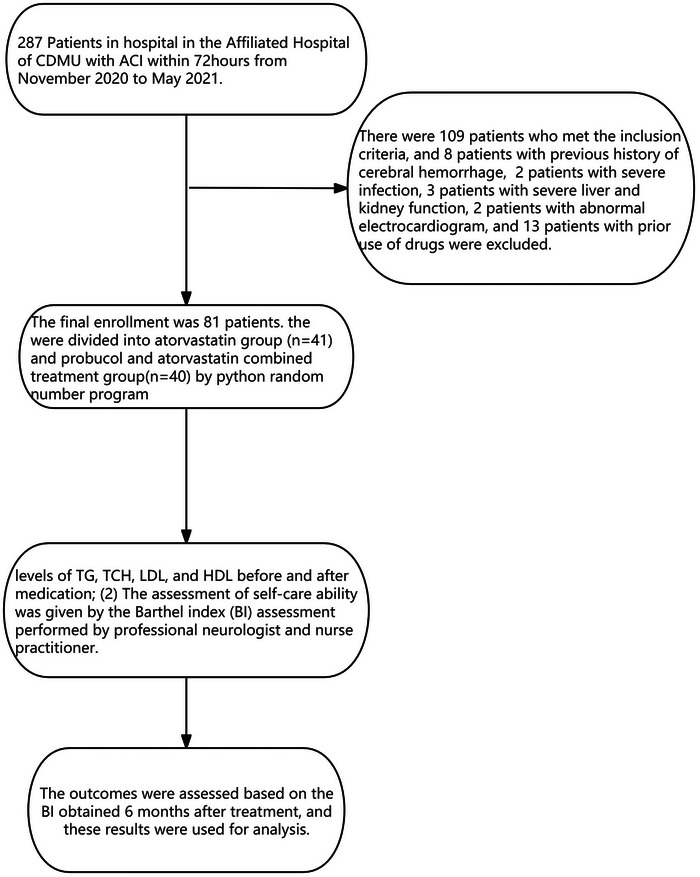
The combination treatment design is depicted.

The following observation indicators were recorded: (1) Levels of triglyceride (TG), total cholesterol (TCH), low‐density lipoprotein (LDL), and high‐density lipoprotein (HDL) before and after medication; (2) The assessment of self‐care ability was given by the Barthel index (BI) assessment performed by a professional neurologist and nurse practitioner. The outcomes were assessed based on the BI obtained 6 months after treatment, and these results were used for analysis. The combination treatment design is depicted in Figure 1.

### 2.3 Baseline Data

The hospital's electronic record system was used to collect general data on the patients, including their sex, age, body mass index (BMI), educational level, and history of cerebrovascular disease, hypertension, diabetes, hyperlipidemia, and heart disease, including atrial fibrillation and coronary heart disease.

### 2.4 Biochemical Data Collection

The biochemical measurements of triglycerides (TG), total cholesterol (TCH), low‐density lipoprotein cholesterol (LDL), and high‐density lipoprotein cholesterol (HDL) were obtained from fasting blood samples. These four markers constitute the standard blood lipid profile and were used to assess changes in lipid metabolism before and after treatment.

### 2.5 Imaging Data Acquisition

All patients underwent plain MRI and diffusion‐weighted imaging sequence scans. The apparent diffusion coefficient value was measured according to Pullicino's rule (Pullicino et al. 1980). The cerebral infarction volume was calculated subsequently.

### 2.6 Neurologic Deficit Function and Cognitive Examination

The participants were assessed by a trained neurologist within three days of admission using the National Institutes of Health Stroke Scale (NIHSS), and BI.

### 2.7 Statistical Analysis

The data was organized, summarized, and analyzed using SPSS 26.0 (Statistical Product and Service Solutions 26.0) statistical analysis software. Descriptive statistics were used for continuous variables following a normal distribution, presented as mean (standard deviation), while count data were described using frequency and percentage [n(%)]. An independent samples t‐test was employed to compare the differences in normally distributed variables between the two groups. A chi‐square test was used to compare the differences in proportions between the two groups. Multivariable generalized estimating equations were utilized to analyze the factors influencing the BI scores. The significance level was set at P < 0.05, indicating statistical significance for differences.

## 3 Results

### 3.1 Basic Characteristics

Based on chi‐square tests, there were no statistically significant differences in terms of sex distribution, vascular history, hypertension, diabetes, and coronary heart disease between the combination treatment and atorvastatin groups (P > 0.05). Two independent sample t‐tests were conducted to analyze the differences between the combination treatment and statin in groups. The results indicated an age, BMI, NIHSS grade, infarct volume, and BI between the two groups (P > 0.05 in all cases). Therefore, the baseline demographic data were comparable between the two groups, as shown in Table 1.

**TABLE 1:** Comparison and analysis of differences in various indicators between the Atorvastatin group and Combination treatment group.

Parameter		Atorvastatin group (n = 41)	Combination treatment group (n = 40)	$\chi^{2}$ value / t value	P value
Age		59.12 ± 9.34	61.58 ± 10.38	−1.119	0.267
BMI		24.78 ± 3.63	25.02 ± 3.23	−0.310	0.758
NIHSS grade		3.05 ± 2.74	2.85 ± 2.38	1.575	0.119
Infarct volume		4.40 ± 12.05	4.41 ± 7.43	−0.003	0.998
BI		76.02±18.78	79.12±18.52	−1.228	0.223
Sex	Male	24 (58.5%)	25 (62.5%)	0.133	0.715
	Female	17 (41.5%)	15 (37.5%)		
History of cerebrovascular disease	Yes	12 (29.3%)	14 (35.0%)	0.305	0.581
	Deny	29 (70.7%)	26 (65.0%)		
Hypertension	Yes	32 (78%)	28 (70.0%)	0.683	0.409
	Deny	9 (22%)	12 (30.0%)		
Diabetes mellitus	Yes	12 (29.3%)	5 (12.5%)	3.433	0.064
	Deny	29 (70.7%)	35 (87.5%)		
Coronary disease	Yes	2 (4.9%)	2 (5.0%)	0.001	0.980
	Deny	39 (95.1%)	38 (95.0%)		

*Notes*: Results are expressed as the mean ± standard deviation or n (%).

*Abbreviations*: BMI, body mass index; NIHSS, National Institutes of Health Stroke Scale.

### 3.2 Blood Lipid Changes Before and After Treatment in the Two Groups

No statistically significant differences were found in the baseline comparison of pre‐treatment lipid profiles between the two groups. However, blood lipid levels showed a decreasing trend in all treated patients. When comparing the results at admission and at six months follow‐up between the groups, there was a statistically significant decrease in the levels of TCH and LDL in both groups. The decrease in TCH was statistically significant in the atorvastatin group (t = 3.241, P = 0.002) and the combination treatment group (t = 2.542, P = 0.013). Similarly, the decrease in LDL was statistically significant in the atorvastatin group (t = 2.136, P = 0.036) and the combination treatment group (t = 2.102, P = 0.039). However, there were no statistically significant differences in the levels of TG and HDL between the groups. Nonetheless, there were statistically significant differences in the levels of TCH (t = 2.204, P = 0.030) and LDL (t = 2.021, P = 0.049) between the two groups, as outlined in Table 2.

**TABLE 2:** Comparative analysis of the lipid profile between the combination treatment and atorvastatin groups (mean ± standard deviation).

Parameter Time group	Baseline				After six months			
	Atorvastatin group	Combination treatment group	t value	*P* value	Atorvastatin group	Combination treatment group	t value	*P* value
TG (mmol/L)	1.90 ± 6.82	1.73 ± 0.92	0.90	0.369	1.73 ± 0.91	1.44 ± 0.61	1.649	0.103
TCH (mmol/L)	4.43 ± 1.03	4.37 ± 1.13	0.27	0.786	4.31 ± 1.16	3.76 ± 1.12	2.204	0.030^ab^
HDL (mmol/L)	1.14 ± 0.33	1.17 ± 0.29	−0.44	0.654	1.17 ± 0.29	1.10 ± 0.32	0.938	0.351
LDL (mmol/L)	2.43 ± 0.80	2.50 ± 0.96	−0.32	0.743	2.48 ± 0.95	2.06 ± 0.94	2.021	0.0049^ab^

***Notes***: a indicates a statistically significant difference between the two groups after treatment; b indicates a statistically significant difference before and after treatment in the same group.

***Abbreviations***: HDL, high‐density lipoprotein; LDL, low‐density lipoprotein.; TCH, total cholesterol; TG, triglycerides.

### 3.3 Changes in BI Scores After 6 Months

Our team re‐evaluated the BI after a 6‐month treatment period. Treatment and rehabilitation have shown improvement in the self‐care ability of patients compared to the baseline during the acute phase. As shown in Table 3, treatment and rehabilitation led to statistically significant improvements in BI scores. In the atorvastatin group, the BI increased from 77.95 ± 17.60 at baseline to 81.48 ± 16.85 after 6 months (P = 0.041). In the combination treatment group, BI scores improved from 79.12 ± 18.52 to 86.13 ± 14.87 (P = 0.004), indicating a more substantial enhancement in self‐care ability.

**TABLE 3:** Changes in Barthel Index (BI) scores before and after treatment.

Group	BI at Baseline (Mean ± SD)	BI at 6 Months (Mean ± SD)	T Value	P Value
Atorvastatin group	77.95 ± 17.60	81.48 ± 16.85	−2.113	0.041 *
Combination group	79.12 ± 18.52	86.13 ± 14.87	−3.101	0.004 **

*P < 0.05 indicates statistical significance.

*P < 0.01 indicates highly significant improvement.

### 3.4 Analysis of Influencing Factors of BI

The findings of Table 4 indicated that the correlation matrix for BI analysis exhibited equal correlation, and the negative binomial function was used for the connection function. Variables with a significance level of P < 0.2 in the univariate analysis were included in the multivariate generalized estimating equation. The results revealed that four variables were included in the model: history of cerebrovascular disease, measurement time, treatment modality, and LDL level. Measurement time was excluded as its effect represents a natural progression over time and would lead to overadjustment. Additionally, to avoid collinearity, treatment modality and LDL level were analyzed in separate models and not interpreted as completely independent predictors. The BI score of patients without a history of cerebrovascular disease was 1.226 times higher compared to those with a history of cerebrovascular disease. At 6 months, the BI score was 1.125 times higher than the baseline score. The combination treatment group had a BI score 1.121 times higher than the atorvastatin group. Patients with elevated LDL did not experience an increase in BI, with a score of 0.916 times higher than the baseline. Additional subgroup analysis revealed that within both the atorvastatin and combination treatment groups, patients with lower post‐treatment LDL levels had significantly higher BI scores at 6 months (*P* < 0.05), indicating a positive correlation between LDL reduction and improvement in self‐care ability. The regression results are summarized in Table 5, showing that history of cerebrovascular disease, treatment duration, treatment modality, and LDL levels were significant factors affecting BI outcomes in both univariate and multivariate models.

**TABLE 4:** Analysis of influencing factors of BI between two group.

		Single factor		Multiple factors	
Metric		*RR*(95%)	*P* value	*RR*(95%)	*P* value
Gender	Male	1			
	Female	0.987(0.883,1.102)	0.810		
History of cerebrovascular disease	Yes	1		1	
	Deny	1.172(1.032,1.332)	0.015	1.226(1.071,1.403)	0.003
Hypertension	Yes	1			
	Deny	0.935(0.814,1.074)	0.341		
Diabetes mellitus	Yes	1			
	Deny	1.078(0.939,1.237)	0.286		
Coronary disease	Yes	1			
	Deny	1.076(0.840,1.379)	0.562		
Measure Time	Baseline	1		1	
	Six months	1.041(1.019,1.064)	<0.001	1.125(1.093,1.157)	<0.001
Treatment modality	Atorvastatin	1		1	
	Combination treatment	1.130(1.016,1.257)	0.024	1.121(1.003,1.254)	0.044
Age		0.997(0.991,1.003)	0.282		
BMI		1.013(0.998,1.029)	0.092	1.002(0.984,1.020)	0.866
TG		1.034(0.971,1.101)	0.303		
TCH		0.982(0.937,1.030)	0.458		
HDL		0.962(0.810,1.143)	0.659		
LDL		0.859(0.712,0.950)	0.018	0.916(0.721,0.948)	0.036

***Abbreviations***: HDL, high‐density lipoprotein; LDL, low‐density lipoprotein.; TCH, total cholesterol; TG, triglycerides.

**TABLE 5:** Generalized Estimating Equation (GEE) Analysis of Factors Affecting BI Score.

Variable	Univariate RR (95% CI)	P value	Multivariate RR (95% CI)	P value
Gender (Female vs. Male)	0.987 (0.883, 1.102)	0.810	—	—
History of cerebrovascular disease (No vs. Yes)	1.172 (1.032, 1.332)	0.015	1.226 (1.071, 1.403)	0.003
Hypertension (No vs. Yes)	0.935 (0.814, 1.074)	0.341	—	—
Diabetes (No vs. Yes)	1.078 (0.939, 1.237)	0.286	—	—
Treatment (Combination vs. Atorvastatin)	1.130 (1.016, 1.257)	0.024	1.121 (1.003, 1.254)	0.044
LDL (per unit decrease)	0.859 (0.712, 0.950)	0.018	0.916 (0.721, 0.948)	0.036

***Notes***: significant values are bolded.

***Abbreviations***: CI, Confidence Interval; RR, Relative Risk.

## 4 Discussion

ACI significantly impacts patients' quality of life due to long‐term functional impairment. (GBDS Collaborators 2021). In this study, we focused on evaluating the effect of combination therapy with probucol and atorvastatin on self‐care ability, measured by the Barthel Index (BI). The results showed that the addition of probucol significantly improved BI scores at 6 months, compared to atorvastatin alone, indicating better recovery of daily function.

In clinical practice, there is currently no universally recognized optimal scale for predicting the outcome of cerebral infarction. This is primarily due to several factors, including the diverse pathogenesis of ischemic stroke, the multitude of risk factors influencing prognosis, the varying composition of risk factors in each scoring tool, the distinct characteristics of the populations in which the models are established, and the differences in the assignment of risk factors. Therefore, it is crucial to explore methods for identifying a more optimal evaluation group within the existing scales and to develop strategies to minimize loss to follow‐up and measurement error during later follow‐up periods. These issues are of utmost importance in order to improve the accuracy and reliability of research findings and enhance the overall quality of patient care. As clinical doctors, we should actively seek ways to address these challenges and implement measures to ensure comprehensive and accurate evaluation of patient outcomes. To address the correlation issues in longitudinal data within non‐independent trial groups, generalized estimating equations (GEE) are more suitable for analysis (Cruz Gutierrez et al. 2023). GEE effectively handles the correlation problems in longitudinal data and provides robust parameter estimates. In our study design, which involved a long follow‐up period, we encountered some missing data. By utilizing GEE to handle repeated measurements in longitudinal data, we successfully addressed the correlation problem and minimized information loss. This approach allowed for a more accurate assessment of changes in patients' self‐care abilities and provided a solid foundation for rational diagnosis and treatment.

Multivariate analysis identified LDL levels and cerebrovascular history as key predictors of BI improvement. Although measurement time showed statistical significance in univariate analysis, its benefit may be mediated by LDL reduction, which was more pronounced in the combination group. These findings may be attributed to the rehabilitation of physical disabilities and the improvement of cognitive abilities observed in our previous study. Our constructed equation suggests that blood lipid levels, particularly LDL levels, are strong predictors of poor prognosis, consistent with previous research (Chen et al. 2020; Herpich and Rincon 2020; Ni et al. 2020). Our subgroup analysis further confirmed this trend within individual groups: patients with greater LDL reductions—whether in the atorvastatin group or the combination treatment group—tended to have higher BI scores. This suggests that the LDL‐lowering effect, especially when enhanced by probucol, plays a crucial role in preserving self‐care ability in ACI patients. Therefore, it is crucial to enhance the management of blood lipids and intervene in the treatment of atherosclerosis. Atorvastatin, an inhibitor of 3‐hydroxy‐3‐methylglutaryl coenzyme reductase, reduces liver synthesis of lipoproteins, promotes the formation of low‐density cholesterol receptors, lowers serum cholesterol levels, regulates blood lipids, and inhibits the activation of peripheral blood macrophages. This, in turn, down‐regulates the levels of inflammatory cytokines, leading to a reduction in inflammatory factors. A previous study has indicated (Hirata 2021) that additional effective drug interventions are necessary when using statins to prevent the recurrence of cardiovascular and cerebrovascular events, thereby reducing the “residual risk” associated with statin use. Probucol, a bisphenol compound, reduces LDL‐C levels independently of LDL receptor activity before the initiation of statin therapy. A recent prospective cohort study conducted by Japanese researchers found (Arai et al. 2022) that probucol increases the risk of recurrent cardiovascular events when added to conventional lipid‐lowering therapy. Our findings are consistent with prior studies suggesting the additive benefit of probucol in cardiovascular risk reduction (Guo et al. 2019). In our study, patients receiving combination therapy achieved greater improvement in BI scores, likely due to enhanced lipid‐lowering and potential neuroprotective effects. Probucol has been shown to effectively lower LDL‐C and may enhance statin efficacy through additional antioxidant mechanisms.

## 5 Limitations

This study has several limitations. First, the sample size was relatively small, which may affect the generalizability of the results. Second, the Barthel Index scoring was conducted by the participating clinical staff, and although trained, some variability may still exist. Third, the study duration was limited to six months; thus, long‐term outcomes remain unknown. Future studies with larger cohorts and extended follow‐up periods are necessary to validate these findings.

## 6 Conclusion

This randomized controlled trial demonstrated that combination therapy with probucol and atorvastatin significantly improved self‐care ability and blood lipid profiles in patients with ACI over a 6‐month period. The BI scores improved more substantially in the combination group, and multivariate analysis confirmed that treatment modality, measurement time, LDL levels, and cerebrovascular history were significant influencing factors.

These findings suggest that combining probucol with atorvastatin may offer enhanced neuroprotective and lipid‐lowering benefits, supporting its potential application in the management of ACI to improve patient outcomes and reduce disability.

## Author Contributions

**Ning Yu**: conceptualization, writing–original draft. **Yajing Li**: supervision, formal analysis, writing–review and editing. **Xue Han**: data curation. **Yuan Sun**: investigation. **Yun Li**: formal analysis. **Yanjun Gao**: conceptualization, supervision, funding acquisition. **Xiaoxuan Zhang**: conceptualization, data curation.

## Ethics Statement

This study has been performed in accordance with the Declaration of Helsinki and was approved by the ethics committee of the Affiliated Hospital of Chengde Medical College (Approval No.: LL047). All participants signed the informed consent form.

## Consent for Publication

The authors have nothing to report.

## Conflicts of Interest

The authors declare that the research was conducted in the absence of any commercial or financial relationships that could be construed as a potential conflict of interest.

### Peer Review

The peer review history for this article is available at https://publons.com/publon/10.1002/brb3.70552

## Data Availability

The raw data supporting the conclusions of this article will be made available by the authors without undue reservation.
